# Reproducibility of Macular Vessel Density Calculations Via Imaging With Two Different Swept-Source Optical Coherence Tomography Angiography Systems

**DOI:** 10.1167/tvst.7.6.31

**Published:** 2018-12-21

**Authors:** Takuhei Shoji, Yuji Yoshikawa, Junji Kanno, Hirokazu Ishii, Hisashi Ibuki, Kimitake Ozaki, Itaru Kimura, Kei Shinoda

**Affiliations:** 1 Department of Ophthalmology, Saitama Medical University, Saitama, Japan

**Keywords:** OCT angiography, vessel density, reproducibility, superficial capillary plexus

## Abstract

**Purpose:**

To evaluate the reproducibility of vessel density calculations using different binarization methods obtained via two commercially available swept-source optical coherence tomography angiography (SS-OCTA) systems.

**Methods:**

Healthy volunteers were imaged using two swept-source optical coherence tomography angiography (SS-OCTA) devices, PLEXElite and Triton. SS-OCTA examinations were performed using a 3 × 3-mm volume scan pattern centered on the fovea. A total of six methods were used for binarization in ImageJ, two global thresholding and four local adaptive thresholding methods. Resultant vessel density values were compared between the instruments and binarization methods. Images for 60 eyes from 30 healthy subjects were assessed by two reviewers who were blinded to the scanning system used.

**Results:**

Twenty-two eyes were excluded due to poor image quality (17 eyes from Triton, 4 eyes from PLEXElite, and 1 eye from both instruments, *P* = 0.003). A final 38 eyes from 23 subjects were eligible for analysis. Each binarization method and instrument led to different median values. The coefficients of variation for vessel density measurements ranged from 0.3% to 2.3% and 0.6% to 4.7% for the PLEXElite and Triton, respectively. Local adaptive thresholding methods revealed higher reproducibility than did global thresholding methods for both devices.

**Conclusions:**

Macular scans with both SS-OCTA instruments showed good reproducibility for vessel density measurements. PLEXElite recorded fewer poor images and had higher reproducibility than did Triton. These findings will inform the selection of proper binarization methods for the clinical detection of vascular diseases affecting the central retina.

**Translational Relevance:**

The reproducibility for macular vessel measurements with SS-OCTA instruments was good. PLEXElite recorded fewer poor images and had higher reproducibility than did Triton.

## Introduction

Optical coherence tomography angiography (OCTA) was recently developed to visualize and measure the retinal microvasculature without the need for invasive intraocular dye injections.^1–4^ OCTA enables the study of both the superficial and deep retinal vessels, including those in the macular region.^1,2,4^

The macula is among the most metabolically active of all human tissues and derives its oxygen supply from multiple retinal capillary plexuses.^5,6^ Approximately one-half of retinal ganglion cell somas are concentrated in the macula.^7,8^ They depend on regional capillary networks to meet their high metabolic requirements. Deficiencies in these networks can result in various diseases, such as diabetic retinopathy, retinal vein occlusion, and glaucoma. For instance, Rao et al.^9^ reported that parafoveal vessel density was significantly lower in glaucoma than in healthy eyes.

At present, two swept-source (SS) OCTA employed in a PLEXElite (PLEX Elite 9000, Version 1.6.0.21130; Carl Zeiss Meditec, Jena, Germany) based on microangiography (OMAG) and Triton (Topcon DRI OCT Triton Swept source OCT; Topcon, Tokyo, Japan) using the so-called OCTA ratio analyses (OCTARA) algorithm are commercially available. While some spectral-domain (SD) OCTA instruments can measure vessel density with built-in programs,^10–17^ studies typically employ the use of public domain ImageJ software (National Institutes of Health, Bethesda, MD) to calculate vessel density with various binarization methods such as automated thresholding,^18^ global and local Otsu,^19,20^ Niblack,^21^ Sauvola,^22^ and Phansalkar.^22,23^ Moreover, few studies have compared instruments^24^ and binarization methods.

Thus, the purpose of this study was to evaluate the reproducibility of vessel density calculations using different binarization methods on the images obtained using two commercially available SS-OCTA systems.

## Methods

### Study Population

This prospective cross-sectional study was approved by the Ethics Committee of Saitama Medical University and conducted in accordance with the tenets of the Declaration of Helsinki. Healthy subjects were included if they were 20 years of age or older, fulfilled the eligibility requirements detailed below, and signed an informed consent form after being made aware of all possible consequences of the study. The study and recruitment occurred between April 2017 and October 2017. Healthy subjects were recruited from the ophthalmology outpatient clinic at Saitama Medical University Hospital (Saitama, Japan). All participants underwent a comprehensive ophthalmic examination, including slit-lamp biomicroscopy, measurement of intraocular pressure (IOP; via noncontact tonometry; Tonoref II, Nidek Co., Ltd., Aichi, Japan), fundus photography (CX-1; Canon Inc., Tokyo, Japan), measurement of axial length and central corneal thickness (Optical Biometer OA-2000; Tomey Corp., Nagoya, Japan), automated visual field (VF) measurement via the Humphrey Field Analyzer (HFA; Carl Zeiss Meditech, Dublin, CA) 24-2 Swedish Interactive Threshold Algorithm, measurement of retinal nerve fiber layer thickness (Spectralis HRA 2; Heidelberg Engineering, Heidelberg, Germany), and macula angiography using two SS-OCTA instruments with PLEXElite and Triton.

Exclusion criteria for all eyes included the following: (1) participant aged under 20 years; (2) reflective error more than +3 diopters (D) or less than −6.0 D; (3) axial length exceeding 26 mm; (4) repeatable measurements of glaucomatous VF damage, defined as a glaucoma hemifield test performance outside normal limits or a pattern with a standard deviation (SD) outside of 95% of normal limits^25^; (5) nerve fiber layer thinning outside of normal limits; (6) evidence of other ocular diseases, including diabetic retinopathy, retinal vein/artery occlusion, age-related macular degeneration, retinal detachment, tilted disc, pseudo exfoliation syndrome, high myopia, and ocular neuropathy; and (7) poor image quality due to motion artifacts or an off-center image, as selected by two blinded examiners according to the criteria described below (qualitative protocol).

### Optical Coherence Tomography Angiography

A 3 × 3-mm OCTA image centered on the fovea was scanned using an SS-OCTA (with PLEXElite and Triton) and the area of superficial retinal vessel density in the macula was calculated.

For PLEXElite images, which featured a central wavelength of 1060 nm, an A-scan rate of 100,000 scans per second, and an axial and transverse tissue resolution of 1.95 and 10 μm, respectively, were used. Prototype OCTA software was used for the acquisition of 3 × 3-mm cubes, with each cube 300 × 300 pixels in size. Angiography images were processed using both phase/Doppler shift and amplitude variation (Optical Micro Angiography), as has been described previously.^26^ The superficial retinal layer (SRL) is defined as from the internal limiting membrane (ILM) to the inner plexiform layer (IPL). For the Triton which features a central wavelength of 1050 nm, a speed of A-scan rate of 100,000 A-scans per second, and an axial and transverse tissue resolution of 8 and 20 μm, respectively. OCT-A software was used for the acquisition of 3 × 3-mm cubes, with each cube composed by 320 × 320 pixels. Angiography image was processed by using amplitude named OCT Angiography Ratio Analysis, which is motion contrast measure using ration method. SRL is defined as from 2.6 μm below the ILM to the 15.6 μm below the IPL. All OCTA scans were performed twice a day for evaluate the reproducibility.

### Qualitative Protocol

Two masked reviewers (YY and HI) reviewed all images independently. As in previous studies,^14,15^ poor-quality scans were excluded from the analyses if any of the following criteria were met: (1) poor-clarity images; (2) weak local signal caused by artifacts such as visual floaters; (3) residual motion artifacts visible as irregular vessel patterns or disc boundaries on the enface angiogram; and (4) images with an off-center fovea. Discrepancies between the two reviewers were resolved by consensus or adjudication by a third experienced reviewer (TS).

### Evaluation of Capillary Signals

The vessel density was calculated for SRL in the macula area. To analyze angiography signals, we performed the global Otsu and Mean as global thresholding methods and local Otsu, Phansalkar, Niblack, and Saubora as adaptive local thresholding methods. These methods were used for binarization algorisms in OCTA images using ImageJ software without noise removal filter to obtain the vascular signals as a white region and digitize this area.^27–30^ Vessel density value was defined as a proportion of an angiography signal in 3 × 3-mm whole macula area.

### Statistical Analyses

All subject characteristics and vessel density values are expressed as the mean ± SD. Intraobserver coefficients of correlation (ICC) and coefficients of variation (CV) are expressed as the mean ± 95% confidence interval (CI).

To evaluate the reproducibility of vessel density measurements between binarization images, ICC and CV were also calculated. We used the paired *t*-tests to compare detected vessel densities between the instruments.

A *P* value less than 0.05 was considered to indicate a statistically significant difference and ICC values over 0.80 indicated almost perfect agreement between every set of two repeated measurements. ICC values below 0.40 indicated poor to fair agreement between the repeated measurements. All statistical analyses were performed using JMP version 10.1 software (SAS Institute Inc., Cary, NC), Stata software version 14 (StataCorp LP, College Station, TX), and SPSS version 25 software (SPSS, Chicago, IL).

## Results

A total of 30 healthy participants were enrolled in this study. From these, a total of 120 images of 60 eyes were acquired. Twenty-two eyes were excluded due to poor image quality (17 eyes from Triton, 4 eyes from PLEXElite, and 1 eye from both instruments; *P* = 0.003 between the devices). Thus, 38 eyes from 23 total subjects were eligible for analysis.

Table 1 summarizes subjects baseline demographics and clinical characteristics. The median subject age was 29 years and the mean spherical error was −2.3 ± 2.4 D. The mean axial length was 24.4 ± 0.8 mm and the mean IOP was 13.4 ± 2.9 mm Hg. Two subjects (5.3%) had a history of smoking and nine subjects (23.7%) had a history of drinking. Table 2 shows vessel density values from the two SS-OCTA instruments and six binarization methods used in the present study. These values differed between the instruments and among the binarization methods. PLEXElite methods led to higher detected vessel density values than did Triton across all binarization methods employed.

**Table 1:**
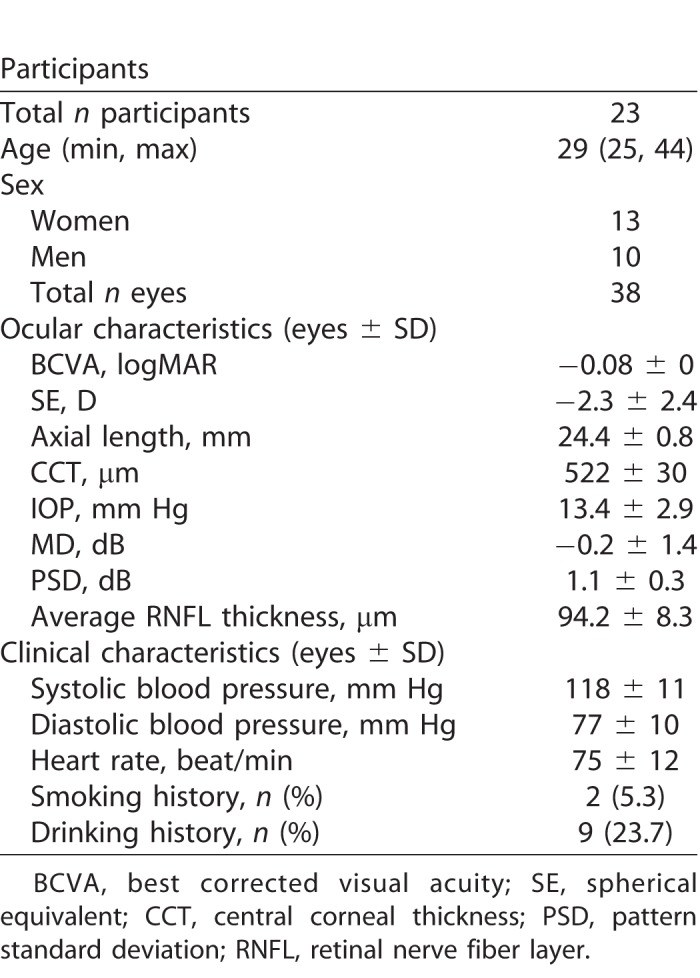
Participant Baseline Characteristics

**Table 2:**
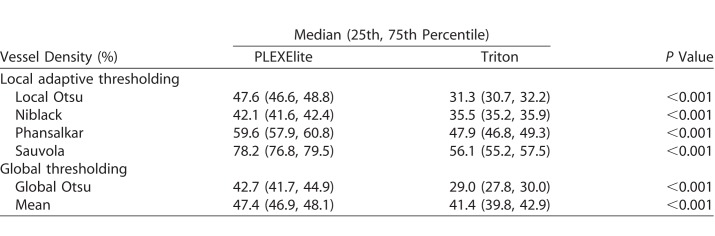
Mean Vessel Density Values

**Figure 1:**
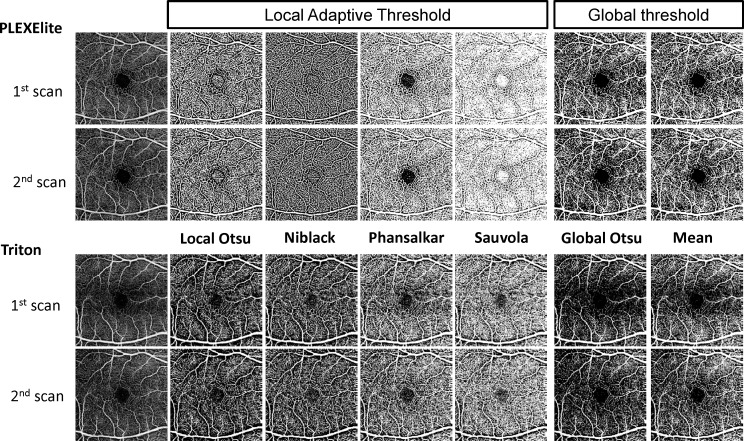
Representative en face scans using PLEXElite and Triton. OCT images (left) and binarized images using local Otsu, Niblack, Phansalkar, Sauvola, and Global Otsu and mean (from left to right).

Figure 1 shows an example OCTA image and binarized images using the two SS-OCTA devices and six different binarization methods. Table 3 shows the ICC and CV values resulting from use of the six binarization methods. PLEXElite led to significantly better CV values than Triton, except for when Phansalkar's method was used (*P* = 0.076).

**Table 3:**
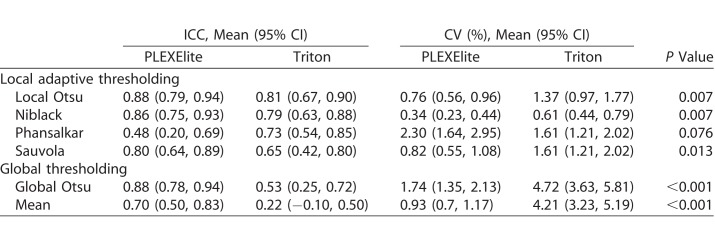
Reproducibility of Various Binarized Methods Between Triton and PLEXElite SS-OCTA Devices

**Figure 2:**
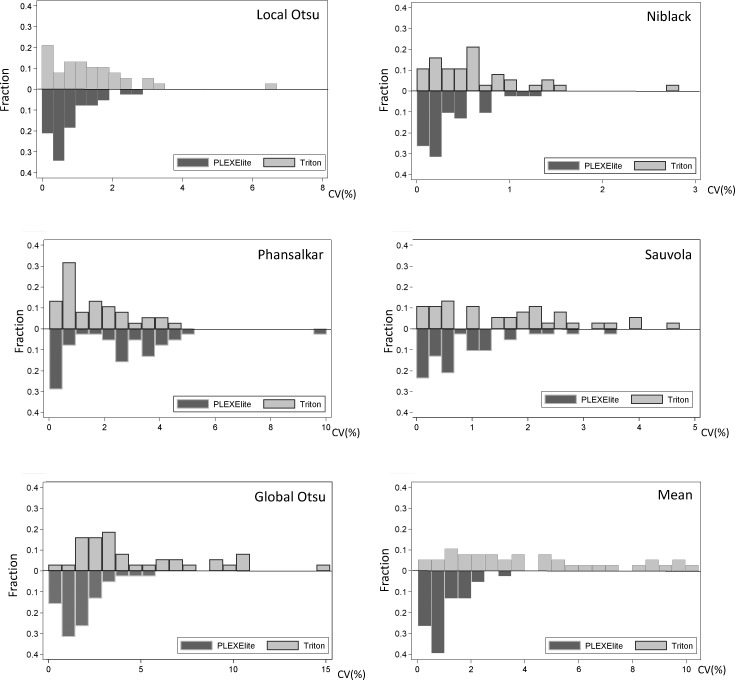
Distribution of CV values for PLEXElite and Triton. Local Otsu (top left), Niblack (top right), Phansalkar (middle left), and Sauvola (middle right) as local adaptive thresholding methods had few outliers in both instruments. Global Otsu (bottom left) and Mean (bottom right) as global thresholding methods were widely distributed in Triton CV values were up to 10% to 15%.

Among the local adaptive thresholding methods, Niblack's method yielded the best CV values across both devices (0.34% for PLEXElite and 0.61% for Triton). In contrast, global thresholding methods yielded worse CV values with Triton (4.72% with Global Otsu's method and 4.21% with Mean method). Figure 2 shows the distribution of CV values derived from PLEXElite and Triton across all binarization methods. While there were few local adaptive thresholding outliers, the data were widely distributed when using global thresholding methods and Triton.

## Discussion

In the present SS-OCTA study, we quantified macular vessel density using several binarization methods and compared reproducibility between two commercially available SS-OCTA instruments across multiple binarization methods to determine the most optimal binarization methodology. Although there have already been several published investigations of OCTA metrics and their reproducibility in healthy subjects, the present report adds to this existing literature in several ways. First, it is the first to investigate the reproducibility of various binarization methods and reveals that local adapting threshold methods are more appropriate when investigating vessel density using OCTA images. Second, the present study is the first to investigate the reproducibility of measures derived from two commercially available SS-OCTA devices. Finally, we confirm here that both SS-OCTA instruments yielded excellent reproducibility of vessel density values across separate trials.

Some studies have attempted to determine the reproducibility of macular vessel density data using SD-OCTA and built-in software. For example, Manalastas et al.^16^ reported that the CV of 3 × 3-mm macula whole image vessel densities in the healthy eye to be 2.5% using the Avanti AngioVue (Optovue Inc., Fremont, CA). Li et al.^31^ similarly reported CVs ranging from 2.4% to 5.9% for perfusion density using the Angioplex (Carl Zeiss Meditec, Jena, Germany). In the present study, CV values for local adaptive thresholding were less than 1% when using PLEXElite and less than 2% when using Triton, values that were better than past SD-OCTA studies had reported.^16,31^ These results suggest that SS-OCTA might have better reproducibility than SD-OCTA due to its higher scan speed, shorter scan time, and longer wavelengths (1050 and 1060 nm) emitted from its light source. Whereas most all commercially available SD-OCT devices use a super luminescent diode (SLD), with a central wavelength of approximately 840 nm as its light source, which is detectable by the human eye, SS-OCT devices use infrared light as their light source, meaning that subjects are unaware of its use during scanning. Given this, the effects of the SS-OCT scanning light on eye and pupil movement, such as miosis, are minimal.

An appropriate image binarization technique, which accounts for uneven illumination, image contrast variation, and poor image resolution, is essential for accurate application of thresholds to an image. Global thresholding methods, including the Otsu and Mean methods, determine a single threshold value for the whole document and then assign each pixel to the foreground or background based on their gray level value and single thresholding characteristics.^27^ Our results demonstrated that global thresholding methods lead to poorer reproducibility than do local adaptive thresholding methods in Triton, even though we excluded more Triton images due to poor quality than we did PLEXElite images. Thus, images obtained via Triton may be more likely to have uneven brightness. In contrast, the local adaptive thresholding methods, including Niblack,^32^ Sauvola,^33^ and Phansalkar's method,^34^ may employ many different adapted values selected according to local area information, and therefore, result in binarized images that are less likely to be affected by naturally uneven brightness. As a result, many recent studies using OCTA have adopted local adaptive thresholding methods. For instance, Agrawall et al.^21^ attempted to use several different image binarization or thresholding techniques including Otsu's, Bernsen's, and Niblack's on SD-OCT images. The authors adopted Niblack's autolocal threshold technique in their choroidal vascularity index study. Rochepeau et al.^22^ selected the Phansalkar method, which is a modification of Sauvola's thresholding method, and deals with darker regions in low contrast images to binarize en face choriocapillaris images obtained via SD-OCTA. The current results were in line with these previous reports, with local Otsu and Niblack's methods demonstrating excellent reproducibility across use in both devices.

Vessel density varies according to the estimation device and binarization method used. Although Munk et al.^35^ qualitatively and quantitatively compared four OCT-A devices, including three different SD-OCTAs and one SS-OCTA and found no significant differences in vessel densities among them, others have reported different results. For instance, Corvi et al.^24^ studied seven different OCTA devices consisting of six SD-OCTAs and one SS-OCTA with one automated thresholding algorithm. Corvi et al.^24^ concluded that comparisons between instruments were nearly impossible and that the set of measurements obtained from the various instruments were not interchangeable. The results presented in the current study were consistent with the Corvi et al.^24^ results that vessel density values differed between two SS-OCTA devices, with the PLEXElite yielding higher values than Triton across all binarized methods. Unfortunately, the exact reason for these discrepancies remains unknown. However, possible explanations for our results may be related to our use of a different algorithm for each set of analyses (OMAG for PLEXElite and OCTARA for Triton). Each algorithm featured different segmentation boundaries between OCTA devices and a different number of B-scans in the 3 × 3-mm image area (300 × 300 pixels for PLEXElite and 320 × 320 pixels for Triton).

There are several limitations to the present study that warrant discussion. First, enrolled subjects were all relatively young and collectively had a narrow range of ages. OCTA metrics can be affected by various factors, such as axial length, refractive error, and age. Further studies using a wider range of ages and larger sample size are needed. In addition, the fact that we excluded many eyes (36.7%) due to poor image quality, leads to some suspicion of a selection bias, which further warrants careful interpretation. Additionally, although there are numerous algorithms for binarization, we selected algorithms in this study according to past reports in the literature. Lastly, the binarization method employed here did not exclude all sources of noise, and some were interpreted as real vascular signals. Thus, the algorithm employed was not perfectly efficient at segmenting blood vessel in OCTA images and room exists for further improvement.

In conclusion, the present study reports good reproducibility of SS-OCTA macula vessel density measurements across both the PLEXElite and Triton devices. The PLEXElite recorded fewer images of poor quality and featured greater reproducibility than the Triton and local adaptive thresholding methods also led to greater reproducibility than did global thresholding methods. Given these findings, studies comparing measurements from different binarization methods and devices should be evaluated with caution. The findings reported here may further help to inform the choices others make about the selection of proper binarization methods and interpretation of findings related to vascular diseases that affect the central retina in clinical practice.
